# Emerging Treatments of Cardiorenal Syndrome: An Update on Pathophysiology and Management

**DOI:** 10.7759/cureus.17240

**Published:** 2021-08-17

**Authors:** Deepak Verma, Amena Firoz, Sameer Krishna Prasad Garlapati, Thanmay Sai Charaan Reddy Sathi, Muhammad Haris, Bibek Dhungana, Barun Ray, Gunjan Shah, Bibek Kc, Palak Paudel

**Affiliations:** 1 Internal Medicine/Family Medicine, Janaki Medical College, Janakpurdham, NPL; 2 Research, California Institute of Behavioral Neurosciences & Psychology, Fairfield, USA; 3 Pediatrics, California Institute of Behavioral Neurosciences & Psychology, Fairfield, USA; 4 Internal Medicine, Andhra Medical College, Visakhapatnam, IND; 5 Internal Medicine, King George Hospital, Visakhapatnam, IND; 6 Internal Medicine, Royal Lancaster Infirmary/Health Education England/ North West, Lancaster, GBR; 7 Internal Medicine, California Institute of Behavioral Neurosciences & Psychology, Fairfield, USA; 8 Internal Medicine, KIST Medical College, Lalitpur, NPL; 9 Internal Medicine, B.P. Koirala Institute of Health Sciences, Dharan, NPL; 10 Internal Medicine, Janaki Medical College, Janakpurdham, NPL; 11 Gynaecology, Bhaktapur Cancer Hospital, Kathmandu, NPL

**Keywords:** cardiorenal syndrome, pathophysiology, management, sglt2 inhibitors, tolvaptan, cardiac resynchronization therapy

## Abstract

Cardiorenal syndrome refers to combined cardiac and renal dysfunction that adversely impacts both organs and is also associated with severe clinical outcomes. The pathophysiology is believed to be multifactorial and complex. Increased central venous pressure and intra-abdominal pressure, overactivation of the Renin-Angiotensin-Aldosterone System (RAAS), systemic illnesses like sepsis, amyloidosis, diabetes are important factors in developing the cardiorenal syndrome. Our review article attempts to review the pathophysiology and treatment aspect of cardiorenal syndrome and explores potential therapeutic strategies that can be adopted for the management.

We searched PubMed, EMBASE, Google Scholar for relevant articles using different keywords and Medical Subject Headings, and finalized 38 articles to be included in our study. Cardiorenal syndrome management aims to eliminate venous congestion and fluid retention, which leads to improved cardiorenal status. This is usually achieved using pharmacologic agents like diuretics, vasodilators, inotropes, angiotensin-converting enzyme inhibitors (ACEIs)/angiotensin II receptor blockers (ARBs), neprilysin inhibitors, and extracorporeal methods like ultrafiltration. The use of therapeutic agents such as sodium-glucose co-transporter 2 inhibitors and tolvaptan (a vasopressin V2 receptor antagonist), and cardiac resynchronization therapy has also been shown to have potential benefits in managing the disease. These agents can be instrumental in the management and require large-scale clinical trials specifically aimed at improving cardiorenal outcomes based on severity and type of cardiorenal syndrome.

## Introduction and background

The symbiotic relationship of the heart and kidney function was recognized in the 19th century by Robert Bright, but the term Cardiorenal Syndrome(CRS) was coined in 2004 [1,2]. CRS is the interplay of acute and chronic cardiac and renal disorders. It has been shown in several studies that patients with heart failure (HF) have prominent kidney dysfunction and vice-versa.

**Figure 1:**
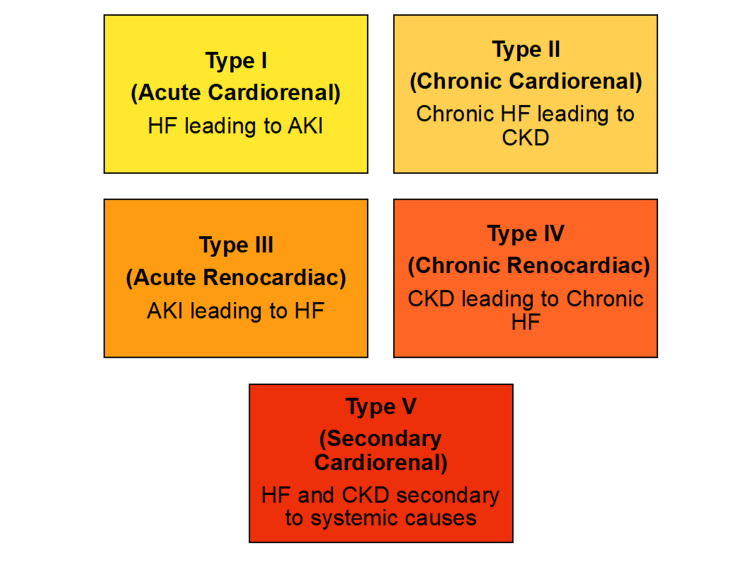
Classification of Cardiorenal Syndrome HF: Heart Failure; AKI: Acute Kidney Injury; CKD: Chronic Kidney Disease

The common risk factors for CRS development are hypertension, diabetes mellitus, atherosclerosis, and medications [1]. The exact pathophysiology of CRS is unclear; however, a few hypothesized mechanisms are increased central venous and abdominal pressure, reduced cardiac output, activation of the Renin-Angiotensin-Aldosterone System (RAAS), and oxidative stress [1]. CRS has been divided into five types depending on onset and primary system involvement; Type I Acute Cardiorenal Syndrome, Type II Chronic Cardiorenal Syndrome, Type III Acute Renocardiac Syndrome, Type IV Chronic Renocardiac Syndrome, and Type V Secondary Cardiorenal Syndrome as seen in Figure 1 [1].

CRS diagnosis is complex; hence, multiple diagnostic tools are used to arrive at an accurate diagnosis, including type. Biomarkers, non-invasive imaging like echocardiography and renal ultrasound, volume assessment, and hemodynamic monitoring devices are diagnostic tools used to assess cardiac and renal function [2].

While diuretics are considered a milestone in managing heart failure, several other strategies have also been used effectively to treat the cardiorenal syndrome, namely, ultrafiltration, vasodilators, and inotropes, and drugs are acting on RAAS pathways like ACEIs/ ARBs, neprilysin inhibitors [2]. Loop diuretics help cause natriuresis, thereby maintaining net negative salt-water balance along with extracellular volume reduction [3,4].

Sodium-glucose co-transporter-2 inhibitors (SGLT2i) are the newer antidiabetic drugs approved by the FDA in 2013 to treat type 2 diabetes [5]. They lower blood glucose levels in an insulin-independent pathway [6]. They exert their effect mainly through blockage of SGLT2 protein in the proximal convoluted tubule (PCT) of the kidney resulting in increased glucose excretion [5,6]. This mechanism of action leads to caloric loss and subsequently creating osmotic diuresis that may be beneficial in lowering blood pressure [6]. They also exert a renoprotective effect and have been found to reduce hospitalization and death from heart failure [7]. Tolvaptan is a selective vasopressin V2 receptor antagonist beneficial in conditions like heart failure and hyponatremia [8]. Cardiac resynchronization therapy is a device therapy used in the treatment of refractory heart failure. It has been shown to improve cardiac function and renal function in patients with CRS [9].

In our study, we review the pathophysiological and treatment aspect of cardiorenal syndrome and also attempt to explore new therapeutic strategies like SGLT2i, tolvaptan, and cardiac resynchronization therapy.

## Review

Discussion

We searched PubMed, EMBASE, Google Scholar for the relevant articles. The keywords cardiorenal syndrome, pathophysiology, management, SGLT2 inhibitors, tolvaptan, and cardiac resynchronization therapy were used to search alone or in combination to yield relevant information. The articles published in languages other than English were not included. Animal trials, duplicated studies, and studies providing insufficient and irrelevant information were excluded from our research.

Pathophysiology of Cardiorenal Syndrome

The pathophysiology of CRS is an unclear one; however, many mechanisms have been postulated. Central venous pressure and intraabdominal pressure (IAH) being important in regulating the blood flow through the kidneys, which preserve their function; an increase in these pressures during heart failure can lead to congestion of blood in the kidneys and impaired renal function. This was illustrated in a study done on 40 heart failure patients, where patients with elevated IAH (8mmHg) also had elevated serum creatinine, demonstrating a correlation between increased IAH and renal impairment [1].

**Figure 2:**
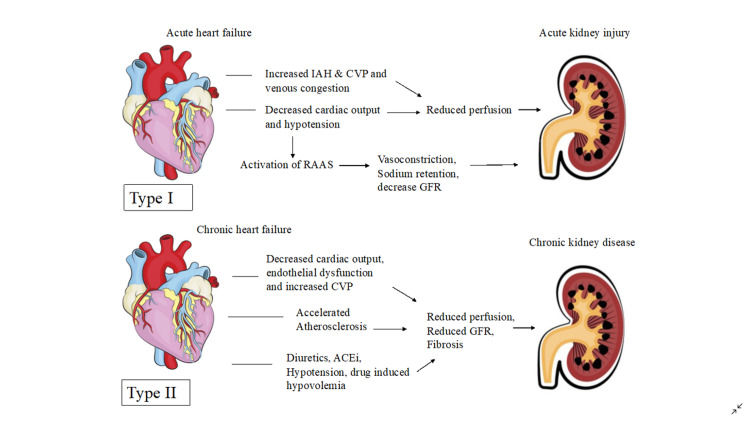
Pathophysiology of Type I and II Cardiorenal syndrome IAH: Intra-abdominal Hypertension; CVP: Central Venous Pressure; RAAS: Renin-Angiotensin-Aldosterone System; GFR: Glomerular Filtration Rate; ACEi: Angiotensin-converting enzyme inhibitors Figure details inspired from Kumar et al. [1]

**Figure 3:**
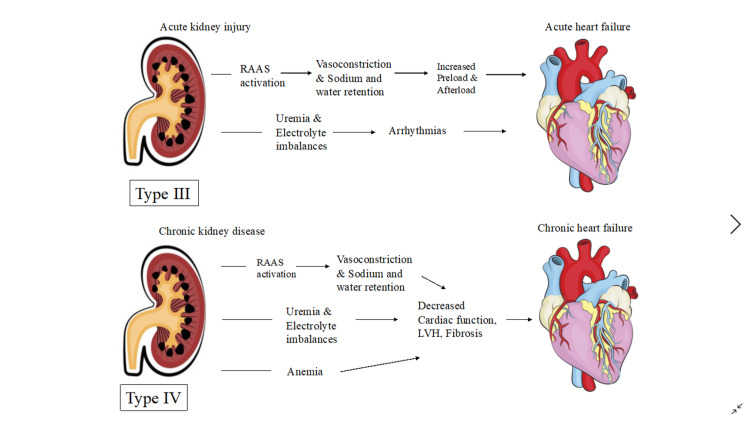
Pathophysiology of Type III and IV Cardiorenal syndrome RAAS; Renin-Angiotensin-Aldosterone System; LVH: Left Ventricular Hypertrophy Figure details inspired from Kumar et al. [1]

The Renin-Angiotensin-Aldosterone System (RAAS) maintains systemic blood pressure and perfusion of visceral organs by systemic vasoconstriction and sodium-water reabsorption, overactivation of this system can lead to renal and cardiac dysfunction [10]. Increased inflammatory markers and free radicals cause endothelial calcification and dysfunction resulting in poor perfusion of the heart and kidneys making them more susceptible to chronic renal and cardiac impairment [1]. Kidneys help maintain electrolyte balance in the body; hence, when impaired, an increase in potassium levels has an adverse effect on the heart, leading to arrhythmias and cardiac arrest. Lastly, systemic diseases like sepsis, diabetes mellitus, amyloidosis, and vasculitis have been shown to trigger all the above mechanisms leading to renal and cardiac impairment and cardiorenal syndrome [10]. The pathophysiology of CRS Type I-IV is illustrated in Figures 2, 3.

Management of Cardiorenal Syndrome 

Initially, reduced cardiac output or lack of forward flow was considered the primary driver of kidney injury; but now, the concept that venous congestion rather than lack of forward flow is the principal cause of worsening renal function is favored. Following are the major treatment strategies used in treating cardiorenal syndrome [2]. 

1. Decongestive therapies - Diuretics, Ultrafiltration

2. Neurohormonal Modulation and Vasodilator and Inotropic therapy

3. RAAS inhibition in chronic CRS - ACEI/ARB, Neprilysin/ Renin-Angiotensin inhibitors, Mineralocorticoid receptor antagonists, Beta-blockers

At the moment, diuretics continue to be the drug of choice for the initial treatment of stable patients with type I CRS. However, diuretics alone have not been shown to improve hard cardiac endpoints [11-13]. Diuretic therapy aims to eliminate clinical evidence of fluid retention, such as elevated jugular venous pressure (JVP) and peripheral edema [14]. It is found that improvement in cardiac function is associated with improved renal function in patients with type I and type II CRS [15]

Although ultrafiltration is done in patients with acute decompensated heart failure (ADHF) and diuretic resistance and/or impaired renal function, it is not an effective therapy for CRS. Studies that address the utility of ultrafiltration in patients with functional diuretic resistance and frequent readmission for ADHF are necessary to see whether clinically and meaningful outcomes can be achieved in these high-risk populations [16,17]

Inotropes have the potential to improve type I CRS by improving cardiac output and reducing venous congestion. Although progress has been made in inotrope and vasodilator therapy, its long-term efficacy in treating ADHF and types I CRS is yet to be demonstrated [2].

ACEIs and ARBs are a standard part of the therapy of HF with reduced ejection fraction, but it is not usually associated with improvement in renal function [2,18,19]. The long-term efficacy of achieving complete suppression of RAAS with an ACEI/ARB is limited by the phenomenon of aldosterone escape, resulting in an increased level of serum aldosterone. Mineralocorticoid receptor antagonists (MRAs), when added to an ACEI/ARB, can provide more suppression of RAAS with potential long-term cardiorenal benefits [20].

Beta-blockers are shown to improve ejection fraction in HF, alleviate symptoms, and prolong survival, but limited data is available on the benefit of beta-blockers in patients with chronic kidney disease (CKD) [14]. Table 1 summarizes the management of cardiorenal syndrome.

**Table 1:** Summary of the Management of Cardiorenal Syndrome ADHF: Acute Decompensated Heart Failure; IV: Intravenous; LVAD: Left Ventricular Assist Device; INTERMACS: Interagency Registry for Mechanically Assisted Circulatory Support; CRS: Cardiorenal Syndrome; RCT: Randomized Controlled Trial; PCWP: Pulmonary Capillary Wedge Pressure

Author	Year	Type of study	No of patients	Purpose of study	Results	Conclusion
Abraham et al. [11]	2005	Observational study	65,180	To compare in-hospital mortality in ADHF in patients receiving IV vasoactive medications.	Decreased mortality in patients receiving nitroglycerine or nesiritide compared to patients receiving dobutamine or milrinone.	The use of natriuretic peptides was associated with a significant reduction in in-hospital mortality compared to positive inotropic therapy in patients with ADHF.
Kirklin et al. [12]	2013	Observational study	4917	To estimate post-implant mortality of LVAD in patients from INTERMACS as per the severity of renal dysfunction.	60% reported mild or no renal dysfunction, 30% with moderate, and 6% with severe renal dysfunction. Increased mortality in severe renal dysfunction.	Pre-implant renal dysfunction was associated with higher mortality. LVAD implants should be considered before CRS advances.
Felker et al. [13]	2011	RCT	308	To assess renal function with administration of diuretics by bolus compared to continuous infusion or at high dose compared to low dose.	No significant difference in change in mean creatinine level. High dose, however, was associated with increased diuresis and favorable outcomes.	No significant difference in patients' assessment of symptoms or change in renal status.
Testani et al. [15]	2010	RCT	336	To study the effect of aggressive decongestion in decompensated heart failure on renal function and survival.	Hemoconcentration was strongly associated with worsening renal status but no change in right atrial pressure and PCWP.	Hemoconcentration was associated with an improved survival rate.
Prosek et al. [16]	2013	Narrative review	-	To assess the role of pharmacologic and extracorporeal methods for hypervolemia in ADHF and CRS.	-	Diuretics and ultrafiltration are effective measures in relieving congestion in ADHF.
Kazory [17]	2013	Narrative review	-	To study the benefits of ultrafiltration in ADHF.	-	Ultrafiltration is an effective measure for the management of ADHF and CRS.
McAlister et al. [19]	2004	Cohort study	754	To study the prevalence of renal insufficiency in heart failure.	Survival outcome was associated with renal status in systolic or diastolic dysfunction.	Renal insufficiency is more prevalent in patients with heart failure and is an independent prognostic factor in diastolic and systolic dysfunction.
Pitts et al. [20]	1999	RCT	822	To study the effect of spironolactone on morbidity and mortality in patients with severe heart failure.	30% reduction in risk of death in patients receiving spironolactone and 35% decrease in hospitalization for heart failure.	In addition to standard therapy, spironolactone reduces the risk of morbidity and mortality in severe heart failure.

Potential Therapeutic Agents for the Management of Cardiorenal Syndrome

SGLT2 inhibitors

Various clinical trials and other studies have demonstrated the potential benefit of the administration of SGLT2 inhibitors. EMPA-REG OUTCOME (Empagliflozin Cardiovascular Outcome Event Trial in Type 2 Diabetes Mellitus Patients-Removing Excess Glucose) trial conducted by Zinman et al. on 7020 patients examined the effect of empagliflozin on cardiovascular morbidity and mortality in type 2 diabetics receiving standard of care. The trial showed composite cardiovascular (CV) events [(CV death, nonfatal myocardial infarction (MI), stroke)] in 10.5% of patients receiving empagliflozin compared to 12.5% of patients on placebo. In contrast, the composite renal outcome ( doubling of serum creatinine level, renal replacement therapy, renal death) was reported to be 1.7% compared to 3.1% in placebo. The study also showed that the drug resulted in a significant reduction of cardiovascular risk factors like weight, waist circumference, uric acid level, and systolic and diastolic blood pressure without increasing heart rate [21]

Another trial conducted by Neal et al., called the CANVAS trial (Canagliflozin Cardiovascular Assessment Study), a set of two sister trials to study the efficacy of canagliflozin on cardiorenal outcomes, showed a composite CV outcome of 26.9 per 1000 patients compared to 31.5 per 1000 of placebo patients and renal outcome of 5.5 versus 9 per 1000 patients in placebo group conducted over 10,142 patients overall. The study attributed the benefits to improved glycemic control, reduction in blood pressure (BP), decreased intra-glomerular pressure, reduced albuminuria, and amelioration of volume overload [22].

Wiviott et al., in DECLARE-TIMI 58 trial ( Dapagliflozin Effect on Cardiovascular Events), showed composite CV outcome in 8.8% of patients receiving dapagliflozin compared to 9.4% of placebo patients and renal outcomes in 1.5% on the drug compared to 2.8% placebo, which included a total of 17,160 patients [23]. CREDENCE trial (Canagliflozin and Renal Events in Diabetes with Established Nephropathy Clinical Evaluation) conducted on 4401 patients showed the composite cardiovascular outcome of 9.9% in patients receiving canagliflozin compared to 12.2% in placebo patients and renal outcomes in 11.1% versus 15.4% in placebo patients. The trial attributed the findings to renin-angiotensin system blockade and reduction in intra-glomerular pressure [24].

A review by Kluger et al. mentioned that the four trials on SGLT2i showed reliable cardiorenal benefits and similar safety profiles. The potential benefits of SGLT2i were found to be due to a reduction in systemic and renal artery stiffness, hyperglycemia, hyperlipidemia, and decreased expression of inflammatory molecules. Increased expression of sodium-glucose co-transporter-1 inhibitors (SGLT1) receptors in cardiomyocytes may have represented a potential pharmacological target for cardio-protection [25]. Effects like lowering of blood pressure and reduced intravascular volume are due to osmotic diuresis, which is thought to be caused due to the inhibition of sodium reabsorption as it is co-transported with glucose [26]. SGLT2i also increases the release of adenosine by increased delivery of sodium to macula densa, which mediates tubulo-glomerular feedback to constrict afferent vessels, thereby reducing intra-glomerular pressure [6]. These groups of drugs also lower urate levels by accelerating renal urate excretion through suppression of the activity of GLUT9b ( Glucose Transporter 9b) [6].

Cardiovascular protection is also partly due to caloric restriction and adaptive ketogenesis. Increased glucose excretion causes a shift to fat utilization, which improves peripheral insulin sensitivity and glucagon secretion, which subsequently releases free fatty acid and increased ketogenesis, enhancing cardiac metabolism. This shift in metabolism lowers renal oxygen consumption alleviating hypoxic stress and thereby slowing the progression of the disease [26].

**Figure 4:**
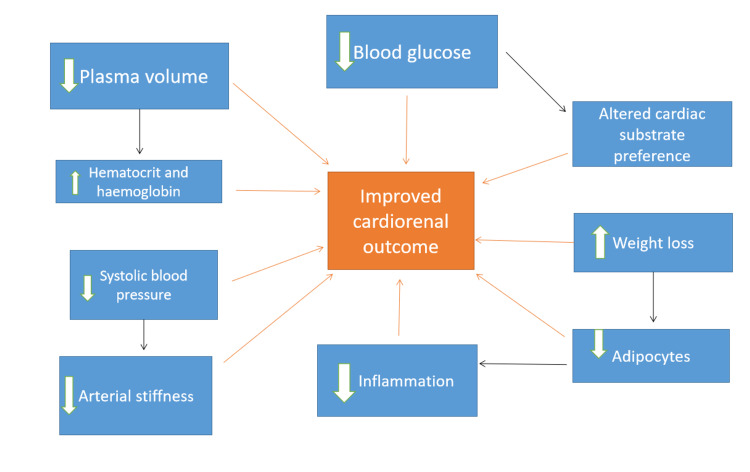
Mechanism of Cardio-renal protection by SGLT2 inhibitors

Besides, Zelniker et al., in their study, mentioned the benefits of SGLT2i in reducing epicardial fat, which might result in decreasing noxious stimuli like leptins and components of RAAS involved in cardiovascular inflammation, and fibrosis. SGLT2i mediated diuresis and natriuresis are also observed mechanisms of renoprotection [27]. The natriuresis effect of the drug in part is attributed to the disruption of functional interaction between SGLT2 and sodium proton exchanger 3 (NHE3) [28]. Figure 4 summarizes the mechanism of cardio-protection by SGLT2i.

Table 2 summarizes the benefits of SGLT2i in CRS. 

**Table 2:** Role of SGLT2i in Cardiorenal Syndrome SGLT2i: Sodium-Glucose co-transporter-2 inhibitor; CKD: Chronic Kidney Disease; RCT: Randomized Controlled Trial;  ESRD: End-Stage Renal Disease; MI: Myocardial Infarction; BP: Blood Pressure; TGF: Tubulo-glomerular Feedback

Author	Year	Type of study	No of patients	Purpose of study	Result	Conclusion
Bailey [6]	2019	Narrative review	-	To study cardiorenal benefits of SGLT2i and lowering of uric acid	-	Improved cardiovascular and renal function and increased excretion of uric acid, thereby slowing the progression of CKD.
Zinman et al. [21]	2015	RCT	7020	To study the effect of empagliflozin on cardiovascular morbidity and mortality in type 2 diabetes.	Decreased rate of cardiovascular morbidity and mortality compared to placebo	Patients with type 2 diabetes receiving empagliflozin had a lower rate of primary composite cardiovascular outcome and death
Neal et al. [22]	2017	RCT	10,142	To study the effect of canagliflozin in cardiovascular and renal events in type 2 diabetes	Lower rate of the primary outcome in patients receiving canagliflozin compared to placebo decreased progression of albuminuria	Lowered risk of cardiovascular events than placebo but increased risk of amputation.
Wiviott et al. [23]	2019	RCT	17,160	To evaluate major adverse cardiovascular events and renal outcomes in patients with type 2 diabetes receiving dapagliflozin.	Lowered rate of cardiovascular death, hospitalization for heart failure, and renal events in patients receiving dapagliflozin compared to placebo.	No significant change in major cardiovascular events but the lowered rate of cardiovascular death and hospitalization for heart failure.
Perkovic et al. [24]	2019	RCT	4401	To determine the effect of canagliflozin on ESRD, doubling of serum creatinine level, and death from renal or cardiovascular causes.	Lowered risk of renal outcome in patients receiving canagliflozin compared to placebo and lowered risk of cardiovascular death, MI, or stroke.	The risk of renal failure and the cardiovascular event was lower with canagliflozin compared to placebo.
Kluger et al. [25]	2019	Systematic review	.	To explore potential determinants of cardiovascular, renal, and safety outcomes in patients receiving SGLT2i	SGLT2i has a lower rate of adverse cardiovascular and renal outcomes.	Baseline renal filtration function and degree of albuminuria are the most important indicator of cardiovascular and renal events.
Kalra et al. [26]	2020	Narrative review	-	To promote rational use of SGLT2i in type 2 diabetes.	-	Reduced cardiac workload, BP mediates cardiovascular protection, and improved lipid profile, whereas renoprotection reduces albuminuria, hypoxic stress, and restoration of TGF.
Zelniker et al. [27]	2018	Narrative review	-	To study cardiorenal effects of SGLT2i in type 2 diabetes	-	Favorable effects on the composite outcome of MI, stroke, and cardiovascular death, reduction in hospitalization for heart failure were observed. It reduced cardiovascular morbidity and mortality with empagliflozin.
Silva Dos Santos et al. [28]	2020	Narrative review	-	To understand the mechanism of cardiorenal protection conferred by SGLT2i.	-	SGLT2i provides cardioprotection by improving glycemia, plasma volume reduction, and reversing proximal tubular neurohumoral dysfunction.

Tolvaptan

Elevated arginine vasopressin levels in heart failure due to activation of RAAS pathway have adverse effects, resulting in deterioration in cardiac function and leading to peripheral vasoconstriction and increased afterload through V1a receptors (Vasopressin 1). Vasopressin V2 receptor stimulation leads to water retention and elevation in preload [8,29]. Tolvaptan has been shown to have a favorable effect on heart failure. It is also helpful in weight reduction, increasing urine output, and correcting serum sodium levels without impacting renal function and serum electrolytes by its action on the neurohormonal pathway in cardiorenal syndrome [8,30].

In EVEREST TRIAL (Efficacy of Vasopressin Antagonism in Heart Failure Outcome Study with Tolvaptan) conducted on 4133 patients, it was found that tolvaptan effectively reduced volume overload and provided symptomatic relief but failed to improve mortality and morbidity [31]. It has a major safety profile and is useful in relieving congestion without any harm on kidney function in patients with volume overload and cardiorenal compromise [32]. Tolvaptan can be beneficial in alleviating congestion and preventing or reducing renal dysfunction by maintaining renal perfusion and avoiding intravascular volume depletion [33]

Cardiac resynchronization therapy

Cardiac resynchronization therapy (CRT) has been used for the treatment of heart failure. It has also improved renal function by improving cardiac output, increased mean arterial pressure, and decreased central venous pressure [34]. In a retrospective cohort analysis conducted by Singal et al. on 260 patients, it was found that renal response improved after CRT in patients with chronic kidney disease (CKD) and congestive heart failure (CHF). Besides, there was a significant reduction in five-year death, transplant, or LVAD ( left ventricular assist device), including patients with stage 4 CKD as well, which was attributed to improved LVEF ( left ventricular ejection fraction), leading to enhanced forward perfusion and decreased venous congestion [35]. CRT also diminishes sympathetic nerve activity decreasing adrenergic tone, which reduces RAAS activity in the long run, explaining improved renal function [36]. Garg et al. also found that CRT may provide a survival benefit in patients with moderate CKD and HF along with improved cardiorenal status [37].

However, a retrospective study including 482 patients reported increased incidence of renal dysfunction at the time of CRT implantation and was usually associated with poor survival outcomes compared to patients with preserved renal function and reported increased mortality in patients with CKD. The authors attributed the findings to a higher prevalence of anemia in CKD [38]. Truong et al. found that one-third of the patients with cardiorenal disease failed to improve renal function with CRT, which might have been due to intrinsic renal disease associated with severe heart failure. The authors attributed the findings to systemic changes leading to cardiac remodeling and worsening glomerular filtration, leading to decreased efficacy of the resynchronization therapy [9].

Limitations 

Most of the available data was inaccessible due to institutional fees or citing confidentiality. There was a lack of large-scale randomized controlled trials aimed at first-hand understanding of cardiorenal syndrome pathogenesis and the effects of different treatments on morbidity and mortality outcomes. Some of the included studies were primarily designed to understand the effects of the therapies mentioned above on heart failure prognosis. However, they did highlight benefits in cardiorenal syndrome and thus call for further research of the topic.

## Conclusions

In this review, we strived to elaborate on the pathophysiology of cardiorenal syndrome with special emphasis on management and newer treatments of cardiorenal syndrome. The impact of RAAS activation on systemic blood pressure combined with oxidative and inflammatory damage to renal vasculature results in cardiorenal syndrome. ACE inhibitors, ARBs, and diuretics countering the effects of RAAS activation were concluded as initial management pillars of these patients. Furthermore, various trials were cited to highlight the efficacy of SGLT-2 inhibitors on morbidity and mortality of these patients when compared to placebo. Tolvaptan, offsetting the effects of elevated vasopressin, has also been demonstrated to have symptomatic benefit in cardiorenal syndrome. Moreover, CRT, which has proven beneficial in heart failure, has also shown improvements in renal function by improving cardiac output.

In our opinion, these newer treatments can prove to be instrumental in the management of cardiorenal syndrome and calls for large-scale trials specifically aimed at understanding these drugs in improving outcomes of patients with cardiorenal syndrome.
